# Establishment and characterization of novel high mucus-producing lung tumoroids derived from a patient with pulmonary solid adenocarcinoma

**DOI:** 10.1007/s13577-024-01060-3

**Published:** 2024-04-17

**Authors:** Miki Iwai, Etsuko Yokota, Yuta Ishida, Takuro Yukawa, Yoshio Naomoto, Yasumasa Monobe, Minoru Haisa, Nagio Takigawa, Takuya Fukazawa, Tomoki Yamatsuji

**Affiliations:** 1 General Medical Center Research Unit, Kawasaki Medical School, Okayama, Japan; 2 Department of General Surgery, Kawasaki Medical School, Okayama, Japan; 3 Okayama Medical Laboratories Co., Ltd., Kurashiki, Japan; 4 Kawasaki Medical School General Medical Center, Okayama, Japan; 5 Department of Medical Care Work, Kawasaki College of Health Professions, Okayama, Japan; 6 Kawasaki Geriatric Medical Center, Okayama, Japan; 7 Department of General Internal Medicine 4, Kawasaki Medical School, Okayama, Japan

**Keywords:** Pulmonary adenocarcinoma, Mucus-producing lung cancer, Invasive mucinous adenocarcinoma, Tumoroids, preclinical cancer model

## Abstract

Among mucus-producing lung cancers, invasive mucinous adenocarcinoma of the lung is a rare and unique subtype of pulmonary adenocarcinoma. Notably, mucus production may also be observed in the five subtypes of adenocarcinoma grouped under the higher-level diagnosis of Invasive Non-mucinous Adenocarcinomas (NMA). Overlapping pathologic features in mucus-producing tumors can cause diagnostic confusion with significant clinical consequences. In this study, we established lung tumoroids, PDT-LUAD#99, from a patient with NMA and mucus production. The tumoroids were derived from the malignant pleural effusion of a patient with lung cancer and have been successfully developed for long-term culture (> 11 months). Karyotyping by fluorescence in situ hybridization using an alpha-satellite probe showed that tumoroids harbored aneuploid karyotypes. Subcutaneous inoculation of PDT-LUAD#99 lung tumoroids into immunodeficient mice resulted in tumor formation, suggesting that the tumoroids were derived from cancer. Xenografts from PDT-LUAD#99 lung tumoroids reproduced the solid adenocarcinoma with mucin production that was observed in the patient's metastatic lymph nodes. Immunoblot analysis showed MUC5AC secretion into the culture supernatant of PDT-LUAD#99 lung tumoroids, which in contradistinction was barely detected in the culture supernatants of NCI-A549 and NCI-H2122 pulmonary adenocarcinoma cells known for their mucin-producing abilities. Here, we established a novel high-mucus-producing lung tumoroids from a solid adenocarcinoma. This preclinical model may be useful for elucidating the pathogenesis of mucus-producing lung cancer.

**Supplementary Information:**

The online version contains supplementary material available at 10.1007/s13577-024-01060-3.

## Introduction

In the 5th edition of the World Health Organization Classification of Lung Tumors, issued in 2021, most pulmonary adenocarcinomas are grouped as invasive non-mucinous adenocarcinoma (NMA) with further histology-based subcategories: Lepidic, acinar, papillary, micropapillary, and solid adenocarcinoma. The subtypes of NMA differ from invasive mucinous adenocarcinoma (IMA); however, they are able to produce mucus, such as pulmonary adenocarcinoma with mucin production [1]. The clinical pathological significance of mucus production in pulmonary adenocarcinoma with mucin production is not well understood, and few studies have been conducted on certain subtypes. MUC5AC, a primary solid component of tumors secreting mucus, has been reported to be associated with a significantly poorer prognosis compared to MUC5AC-negative tumors [2], emphasizing the need to elucidate the underlying pathophysiology. However, there is a limited number of functional preclinical models of pulmonary adenocarcinoma with mucin production [3, 4].

Recently, organoid research has been conducted using pluripotent stem cells, e.g., embryonic stem cells (ESCs), induced pluripotent stem cells (iPSCs), biopsy samples, and excised organs [5, 6]. Organoids are anatomically and functionally similar to organs in vivo, making it possible to analyze life phenomena that were previously difficult [7]. In the field of cancer research, organoids have been established from various kinds of malignant tumors as new preclinical models that can replace cell lines and genetically engineered mice [8, 9]. The term tumoroid has been used as a generic term for organoids derived from malignant tumors, including brain tumors and sarcoma, as well as cancer, in contrast to organoids derived from normal cells and tissues. Tumoroid was used for organoids derived from lung cancer in this study [10–14]. So far, only a handful of preclinical tumoroid models of adenocarcinoma with mucin production have been reported [15, 16]. Moreover, there are no reports on MUC5AC production in cancer organoids of invasive non-mucinous adenocarcinoma (NMA), highlighting the need for the development of excellent preclinical models to elucidate the pathogenesis. In the present study, we established lung tumoroids, PDT-LUAD#99, from the pleural effusion of a patient with mucus-producing solid adenocarcinoma. We further analyzed the genomes of these tumoroids and the pathology of xenografts established from these cells to evaluate the usefulness of these tumoroids as a preclinical model.

## Materials and methods

### Cell lines and culture conditions

NCI-H358, NCI-A549, and NCI-H2122 pulmonary adenocarcinoma cells harboring a *KRAS* mutation and NCI-H3255 pulmonary adenocarcinoma cells harboring the *EGFR*^*L858R*^ mutation were obtained from the American Type Culture Collection (Manassas, VA) and were grown as monolayers in RPMI 1640 (NCI-H358, NCI-H2122, and NCI-H3255) or DMEM (NCI-A549) supplemented with 10% heat-inactivated fetal bovine serum and 100 μg/ml of streptomycin and 100 units/ml of penicillin. All cells were grown under 5% CO_2_ at 37 °C, were authenticated by using short tandem repeats (STRs), and were routinely tested for mycoplasma using a TaKaRa PCR Mycoplasma Detection Set (Takara Bio, Inc., Otsu, Japan).

### Patient-derived tumoroid culture

Patient-derived lung adenocarcinoma (LUAD) tumoroids, PDT-LUAD#19, PDT-LUAD#99, and PDT-LUAD#119, were generated using tumoroid culture systems, as previously described [10]. The research protocol received approval from the Ethics Committee of the Kawasaki Medical School, with the assigned reference number 3171‐5. STR profile analysis was performed in PDT-LUAD#119 tumoroids to investigate genomic stability through future passages with authentication. The patient who participated in the present study signed an informed consent form approved by the responsible authority.

### Next-generation sequencing, Sanger sequencing, quantitative real-time polymerase chain reaction (q-PCR), and  fluorescence in situ hybridization

Next-generation sequencing, including whole exome sequencing and RNA-seq, along with fluorescence in situ hybridization (FISH), was carried out following previously established procedures [10]. MUC5AC mRNA expression was confirmed using a StepOnePlus Real-Time PCR system, with a specific probe for MUC5AC (assay reference: Hs00873651_mH).

Sanger sequencing was performed by Eurofins Genomics K. K. (Tokyo, Japan) using the primers for *TP53* Exon 4: 5′-CAAGCAATGGATGATTTGATGCTGTC-3′ and 5′- TAGGTTTTCTGGGAAGGGACAGAAGATG-3′, and for TP53 Exon 7: 5′- GACAGAGCGAGATTCCATCTCAAAAA-3′ and 5′- ATGAGAGGTGGATGGGTAGTAGTATGGAA-3′.

### Immunoblot analysis, immunohistochemistry, periodic acid–Schiff staining, and Alcian blue staining

Immunoblot analysis and immunohistochemistry were conducted following previously established protocols [10]. The primary anti-MUC5AC antibody (45M1) was obtained from Thermo Fisher Scientific (Rockford, IL, USA), anti-NKX2-1 antibody (8G7G31) was purchased from DAKO (Carpinteria, CA, USA), and anti-HNF4A antibody (H-1) was obtained from Santa Cruz Biotechnology (Santa Cruz, CA, USA). MAC5AC from tumoroids was detected by plating cells at 2.5 × 10^5^ cells per well in a 24-well cell culture plate with 20 μl of basement membrane extract type 2 (BME type 2, R&D Systems, Minneapolis, MN, USA). Following this, the culture medium was replaced with 200 μl of Advanced DMEM/F12 (Thermo Fisher Scientific) without additives, and tumoroids were cultured for 24 h. For the detection of secreted MAC5AC from cell lines, cells were plated at 5 × 10^5^ cells per well in a 6-well cell culture plate. The next day, the medium was replaced with 500 μl of Advanced DMEM/F12, and cells were cultured for 24 h. Subsequently, the supernatant was collected, and after measuring protein concentrations, 10 μg of protein was subjected to immunoblot analysis.

Periodic acid–Schiff (PAS) staining was performed by incubating the slides in 1% periodic acid solution for 15 min and rinsing with running water for 2–3 min. Subsequently, the slides were immersed in Schiff reagent for 20 min, rinsed with a sulfurous acid solution for 1 min and repeated three times, then 2 min and repeated three times, and then rinsed with running water for 10 min. The slides were then counterstained with hematoxylin, dehydrated, and cover-slipped with a mounting medium. Alcian blue staining was performed with a 3 min incubation in hydrochloric acid solution (0.1 N), followed by a 20 min incubation in Alcian blue solution at pH 1.0 (Muto Pure Chemical. Tokyo, Japan). Slides were incubated with a hydrochloric acid solution (0.1 N) for 1 min, repeated this process three times, and then rinsed with running water for 1 min. This was followed by a 2 min immersion in hematoxylin, dehydration, and coverslipping with mounting media.

### Xenograft inoculation of lung tumoroids

Cells from lung tumoroids PDT-LUAD#99 (5 × 10^6^ cells) were dissociated using TrypLE™ Express Enzyme (Thermo Fisher Scientific), mixed with 50 μl of basement membrane extract type 2 **(**BME type 2, R&D Systems, Minneapolis, MN, USA) and subcutaneously injected into 5-week-old NOD/Shi-*scid*/IL-2Rγ^null^ (NOG) mice (Charles River Laboratories Japan, Atsugi, Japan). The mice were sacrificed when the diameter of the subcutaneous tumor reached 15 mm. The period from initiation of the xenografts to the point of euthanasia was around eighty days. All studies were approved by the animal research committee of Kawasaki Medical School (Reference Number: 23‐047). The care and use of animals were conducted in accordance with the committee regulations.

## Results

### Clinical presentation of a patient with parental lung cancer of PDT-LUAD#99

**Fig. 1:**
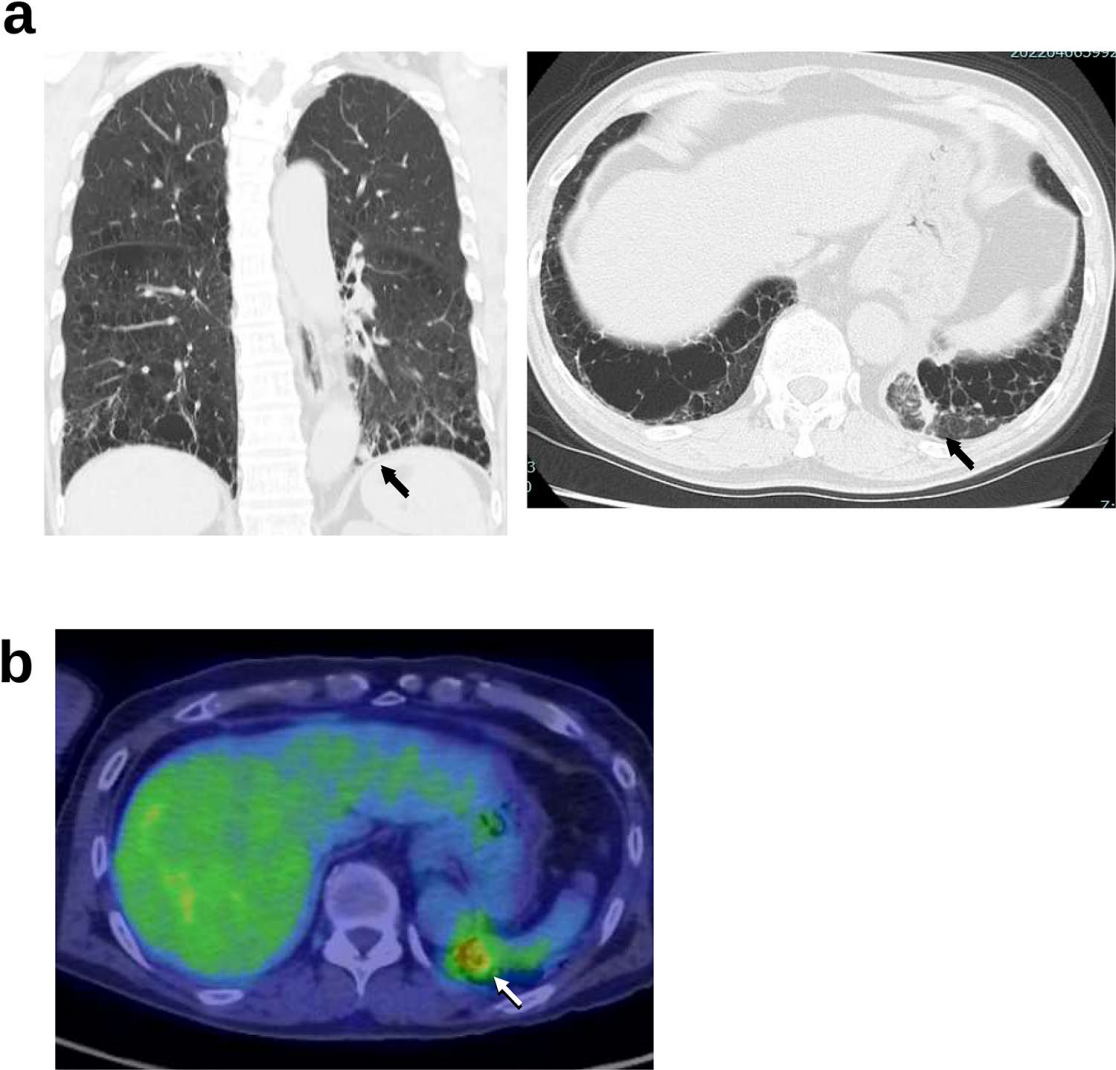
Clinical findings. Computed tomography revealed a nodule with the greatest diameter of 11 mm in the left lower lobe (**a**, black arrow), and positron emission tomography detected an accumulation of fluorodeoxyglucose in the same area (**b**, white arrow)

The patient who was the source of the tumoroids used in the xenografting studies was a 72-year-old male ex-smoker referred to our institution because of an abnormal shadow on chest radiography scans and increased serum carcinoembryonic antigen level (CEA>190 ng/ml). After a thorough examination, the patient was suspected to have lung cancer (cT1bN0M0, Fig. 1a, b), and the left lower lobectomy was considered. However, rapid intraoperative cytology detected malignant cells in the pleural effusion; only the pulmonary ligament lymph nodes (station 9) were excised for sampling, and the chest was closed.

### Postoperative pathological findings

**Fig. 2:**
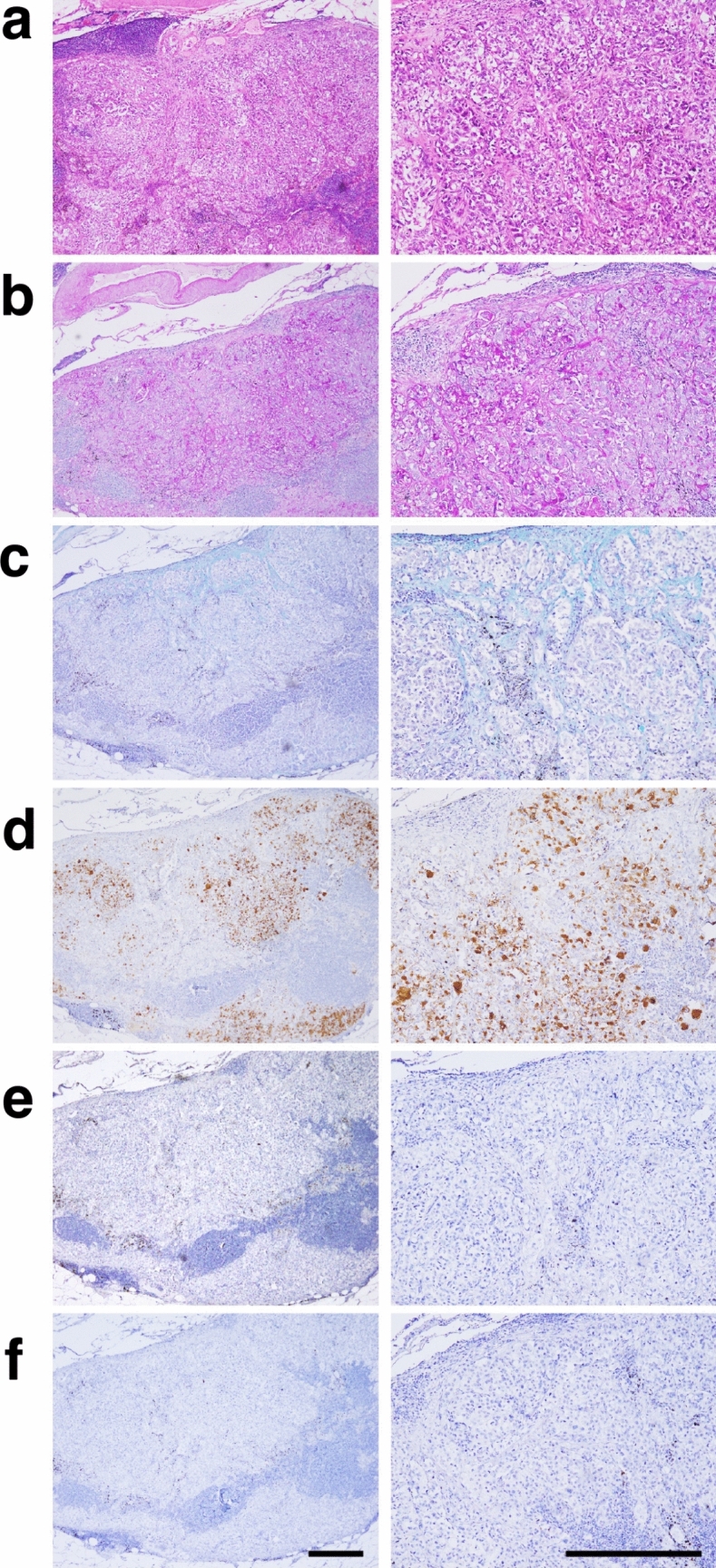
Pathologic features of the lymph node metastases. **a** Hematoxylin-and eosin-stained tissue section of the nodal metastatic tumor. The distinctive pathological features of solid adenocarcinoma were observed. **b** Staining with PAS and **c** Alcian blue revealed cytoplasmic and extracellular mucin in the lung cancer tissue. **d** Immunohistochemical staining showed MUC5AC expression in the lymph node metastases. **e** Limited NKX2-1 and **f** HNF4A expression was observed in the immunohistochemical analysis. Scale bar, 100 μm

Histologic examination revealed lymph nodes extensively replaced by sheets of poorly differentiated adenocarcinoma (Fig. 2a) (pTxN2M1a, stage IVa). Nodal metastatic tumors showed extensive patchy for diastase-resistant PAS positivity and more focal Alcian blue staining, along with limited MUC5AC by immunohistochemistry (Fig. 2b–d). In contrast, expression of the lung tissue markers NKX2-1 and HNF4A was not detected (Fig. 2e, f).

### Establishment of tumoroids from cells of a malignant pleural effusion

**Fig. 3:**
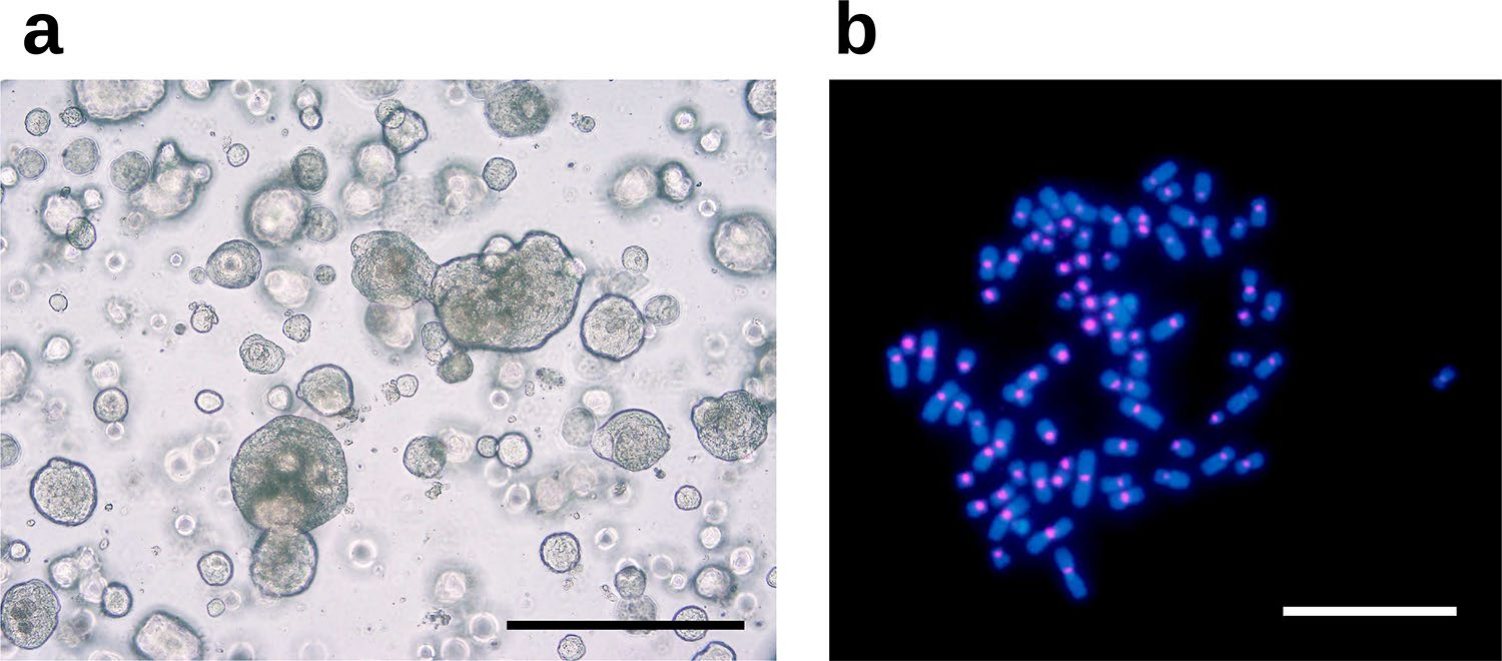
Patient-derived tumoroids: PDT-LUAD#99 tumoroids derived from a patient with pulmonary solid adenocarcinoma. **a** Representative bright-field microscopy images (left ternate columns) of PDT-LUAD#99 lung tumoroids established with different media. Scale bar, 500 μm. **b** A metaphase FISH image (right column) of PDT-LUAD#99 lung tumoroids. Scale bar, 20 μm

In this study, we successfully generated PDT-LUAD#99 lung tumoroids from malignant pleural effusion of a lung cancer patient described as above, and karyotyping using FISH with an alpha-satellite probe demonstrated that PDT-LUAD#99 lung tumoroids harbored aneuploid karyotypes (2*n* = 80), indicating that they were successfully established from lung cancer (Fig. 3a, b). The tumoroids could be cultured continuously for at least 11 months.

### Xenografts derived from the established tumoroids mimic the pathologic features of the lymph node metastases

**Fig. 4:**
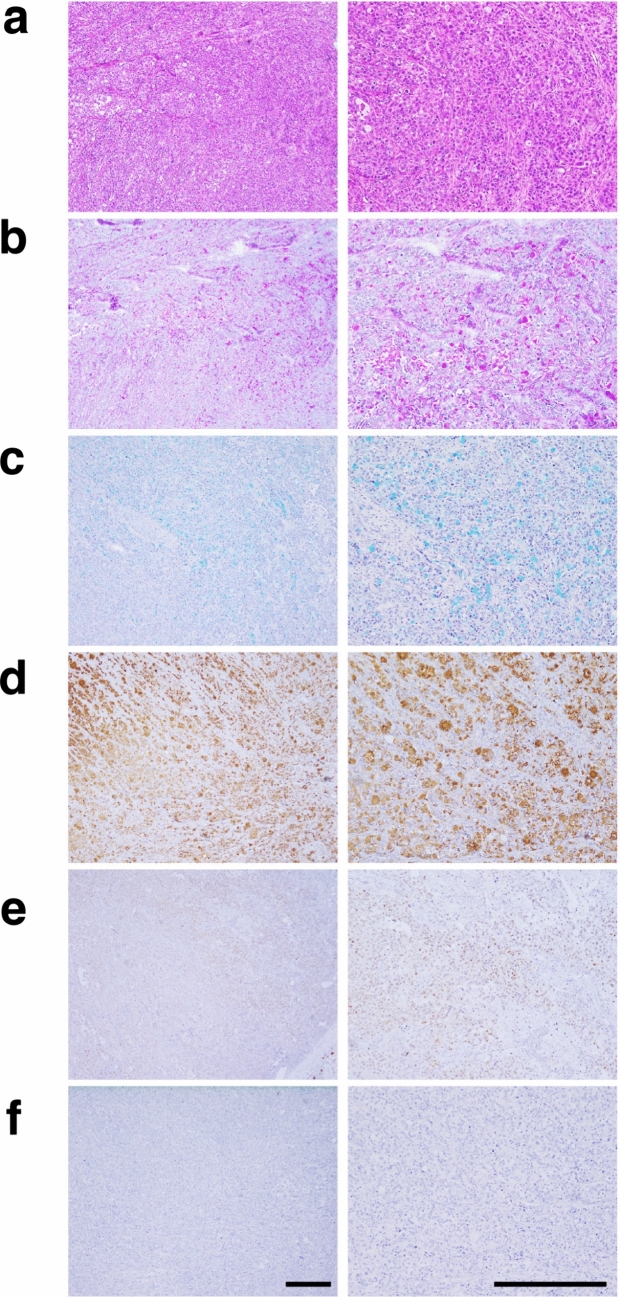
Xenografts derived from PDT-LUAD#99 lung tumoroids recapitulate the pathological features of the lymph node metastases. **a** Hematoxylin and eosin-stained tissue section of the xenograft. The distinctive pathological features of solid adenocarcinoma can be observed. **b** Staining with periodic acid–Schiff with diastase digestion and **c** Alcian blue revealed cytoplasmic and extracellular mucin in the xenograft derived from PDT-LUAD#99. **d** Immunohistochemical staining shows MUC5AC expression in the xenograft from PDT-LUAD#99. **e, f** Limited NKX2-1 and HNF4A expression is observed in the immunohistochemical analysis. Scale bar, 100 μm

Next, to study whether the established tumoroids could recapitulate the pathological features of pulmonary adenocarcinoma observed in lymph node metastases in vivo, we established xenografts in NOG mice by subcutaneous inoculation of PDT-LUAD#99 lung tumoroids. Pathologic examination of these xenografts demonstrated solid, poorly differentiated adenocarcinoma (Fig. 4a), with extensive PAS, Alcian blue, and MUC5AC positivity (Fig. 4b, c, d). HNF4A expression was not observed in the tumors (Fig. 4f). These findings indicated that PDT-LUAD#99 was able to form xenografts, reflecting the patient's lung cancer tissue. In contrast, unlike in the nodal metastatic tumors, NKX2-1 expression was detected in the xenograft (Fig. 4e).

### Whole exome and transcriptome sequencing analysis of the established solid adenocarcinoma tumoroids

**Fig. 5:**
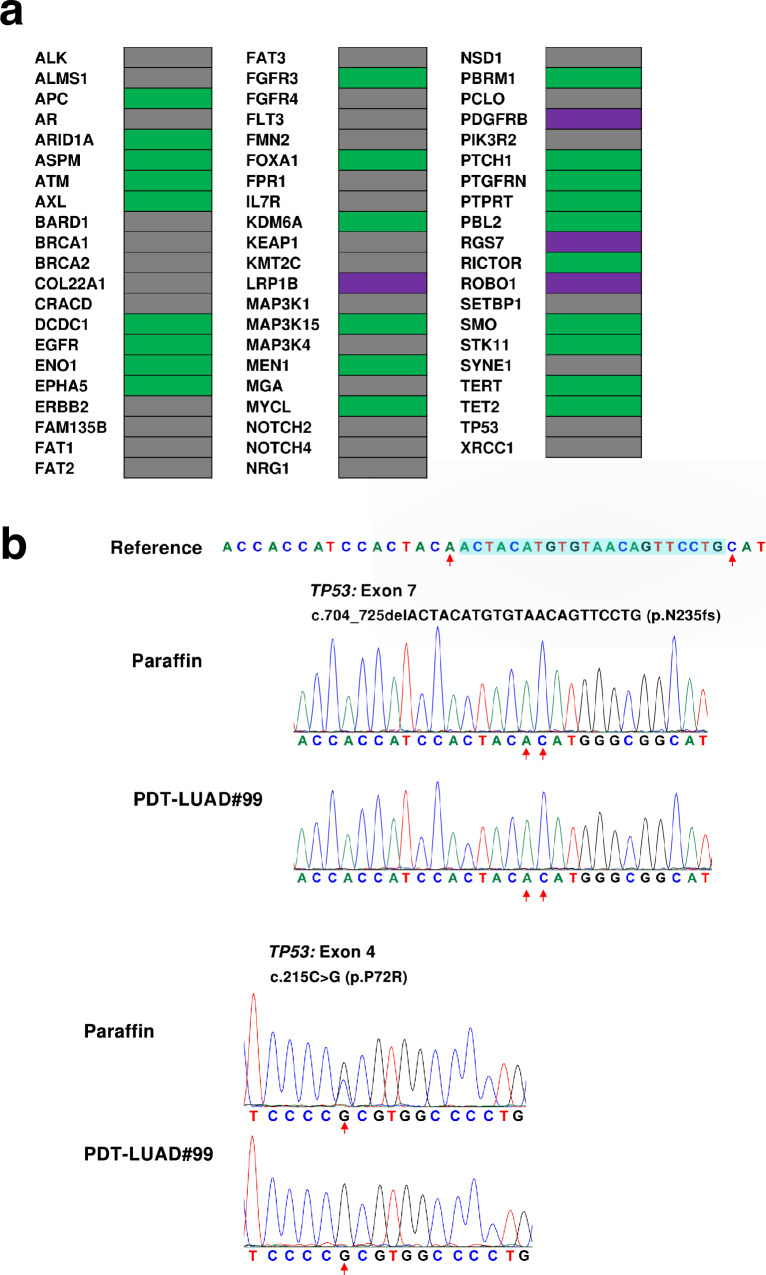
PDT-LUAD#99 lung tumoroids harbor the *TP53* pathogenic mutations detected in the lymph node metastases. **a** Heatmaps showing principal lung cancer genetic variants in PDT-LUAD#99 lung tumoroids. **b** Sanger sequencing confirmed the *TP53* biallelic pathogenic mutations (c.704_725delACTACATGTGTAACAGTTCCTG p.N235fs and c.215C>G, p.P72R) detected in PDT-LUAD#99 lung tumoroids in the genomic DNA derived from the nodal metastatic tumors

To analyze the mutations in PDT-LUAD#99 lung tumors, we conducted whole exome-seq and RNA-seq with genomic DNA and RNA isolated from the tumoroids. Two types of *TP53* pathogenic mutations (c.704_725delACTACATGTGTAACAGTTCCTG p.N235fs and c.215C>G, and p.P72R) were observed in PDT-LUAD#99 lung tumoroids; however, no *KRAS*, *ERBB2,* or *BRAF* mutations, which are frequently observed in IMAs [17], were observed. These *TP53* mutations were also detected in the extracted genomes of the lymph node metastases and lung cancer cells in the malignant effusion of the patient (Fig. 5a, b). Seven *RP11* fusion genes and three *NRIP1* fusion genes were detected, while no *NRG1*, *ERBB4*, *BRAF*, or *RET* fusion genes, which are occasionally found in *KRAS* mutation-negative IMAs, were detected (Table 1). We also performed a STR profile analysis of PDT-LUAD#99 lung tumoroids to investigate genomic stability through future passages (Supplementary Fig. S1).

**Table 1:** Fusion genes detected in PDT-LUAD#99 by RNA sequence

Fusion
EPT1–FAM179A
NRIP1–AF127936.7
PRKCH exon12- RP11-47I22.3 exon2
PR11-123O10.4–GRIP1
PR11-155G15.2–RP11-1E3.1
R11-96H19.1–RP11-446N19.1
NDUFA9 exon8–B4GALNT3 exon2
PR11-494M8.4–OVCH2
PR11-380O24.1–SRGAP3
PR11-120D5.1–MID1

### PDT-LUAD#99 tumoroids secrete more MUC5AC into the culture supernatant than do other lung tumoroids and pulmonary adenocarcinoma cell lines that we examined

**Fig. 6:**
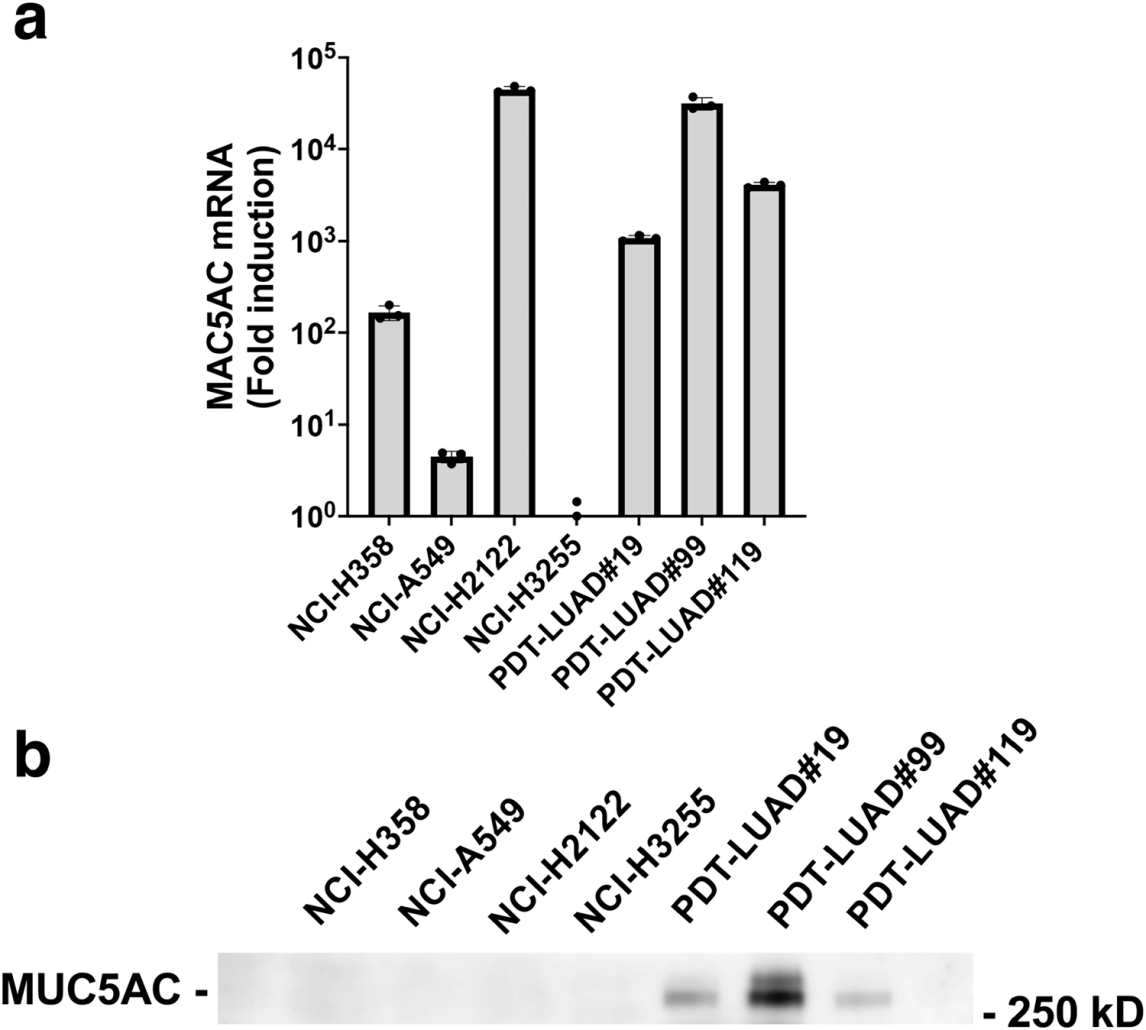
Comparison of mucus production capacity in PDT-LUAD#99 tumoroids and other preclinical lung cancer models. **a** Detection of MUC5AC mRNA expression by q-PCR in the indicated kinds of cells and tumoroids. The displayed data illustrates the fold induction in relation to the mRNA expression of NCI-H3255 cells. Results are mean ± SD of triplicates for each group. **b** Immunoblot analysis showed secreted MUC5AC in the culture supernatant of three kinds of pulmonary adenocarcinoma tumoroids

We compared the mucus-producing capacity of PDT-LUAD#99 tumoroids with that of other preclinical lung cancer models (lung cancer cells or tumoroids). q-PCR analysis demonstrated strong *MUC5AC* mRNA expression in the *KRAS*^*G12C*^ mutant NCI-H2122 pulmonary adenocarcinoma cells, which are known to be mucus-producing [4], and in PDT-LUAD#119 tumoroids. Lower *MUC5AC* mRNA expression was observed in NCI-H358 and NCI-A549 pulmonary adenocarcinoma cells harboring *KRAS*^*G12C*^ and *KRAS*^*G12S*^*,* respectively, and in PDT-LUAD#19 and PDT-LUAD#119 tumoroids. Low expression was also observed in NCI-H3255 cells harboring the *EGFR*^*L858R*^ mutation (Fig. 6a).

Next, we detected secreted MUC5AC in culture supernatants from lung cancer cells and lung tumoroids by immunoblotting. Secreted MUC5AC was clearly observed in the culture supernatant of PDT-LUAD#99, while less of this protein was observed in the culture supernatants of PDT-LUAD#19 and PDT-LUAD#119. Interestingly, limited secreted MUC5AC was detected in all types of lung cancer cells used, including NCI-H2122 cells, in which *MUC5AC* mRNA expression was detected by q-PCR (Fig. 6b).

## Discussion

IMA of the lung accounts for 3–5% of pulmonary adenocarcinomas and is a unique subtype [18, 19]. From a pathological perspective, IMAs exhibit a distinctive morphology with either goblet or columnar cells, with a notable abundance of intracytoplasmic mucin. It has been reported that more than 70% of IMAs harbor *KRAS* mutations, while 6% have *ERBB2* mutations [17]. In some cases that lack *KRAS* mutations, oncogenic fusion genes are present, with *NRG1* being the most frequently involved [20]. In our case, the malignant pleural effusion was viscous, suggesting that the patient had mucus-producing lung cancer. However, the pathological findings revealed that, in the patient's metastatic mediastinal lymph nodes, no tumor cells exhibited a goblet or columnar morphology. The diagnosis was a poorly differentiated solid adenocarcinoma rather than an IMA. Whole exome sequencing showed that PDT-LUAD#99 lung tumoroids harbored *TP53* mutations but not *KRAS* or *ERBB2* mutations and no obvious oncogenic fusion genes were found by RNA-seq, which is consistent with an invasive non-mucinous adenocarcinoma. PAS, Alcian blue, and MUC5AC staining were extensively positive in xenografts derived from PDT-LUAD#99 lung tumoroids. Furthermore, secreted MUC5AC in the culture media of the tumoroids was more abundant than that in mucus-producing NCI-H2122 and NCI-A549 cells [3, 4], suggesting that PDT-LUAD#99 tumoroids display high mucus production. Although NCI-H2122 cells showed higher *MUC5AC* mRNA expression than PDT-LUAD#99 cells, limited secretion of MUC5AC was observed in the culture supernatant. This suggests that three-dimensional (3D) organoid culture may reproduce the mucin secretion ability of the patient’s tumor, whereas two-dimensional (2D) culture may lead to loss of this function [21, 22]. In contrast, Alcian blue staining in the patient's metastatic lymph nodes was partially positive, and MUC5AC expression was limited. In addition, NKX2-1 expression was observed in the established tumoroids; however, limited expression was observed in the lymph node metastases. The most important reason for the discrepant expression pattern could be that the small deposit of metastatic carcinoma in the examined lymph node may have represented an evolved subclone that could be poorly representative of the primary lung tumor or PDT-LUAD#99 was established by clonal selection using culture media or growth factors.

Tumoroids are less expensive to establish and can be produced more rapidly than genetically engineered mouse models and PDXs [23, 24]. Compared with cell lines generated by 2D culture systems, tumoroids exhibit stable genomes and are well-suited for the analysis of primary tumors [25, 26]. In addition to this, tumoroids, by and large, retain most pathologic features of their parental tumors and are expected to be useful preclinical cancer models for the development of novel cancer treatment strategies. Targeted therapies based on molecular subtypes of lung cancer have advanced [27, 28], at the same time as genomic analysis of pathological subtypes of lung adenocarcinoma is progressing.[29]. The establishment of tumoroids derived from different molecular and morphologic subtypes of pulmonary carcinoma promises to lead to a greater understanding of the pathophysiology underlying different tumor types, as well as to the development of effective treatment strategies tailored to individual patients. Clinicopathological features and genomic analysis of IMAs have been investigated to date; however, less attention has been paid to the pathogenesis of NMA with mucin production. The prognosis of patients with these types of tumors is poor [30, 31]; thus, the development of these preclinical models may be useful for investigations into new therapeutic strategies. A limitation of the study is that we have established only one type of tumoroid derived from one patient with NMA with mucin production. More preclinical tumouroid models of pulmonary adenocarcinoma with mucin production should be established for detailed analysis in the future.

### Supplementary Information

Below is the link to the electronic supplementary material.

Supplementary file1 (TIF 982 KB)

## Data Availability

Raw sequencing data for the exome sequence are available under controlled access at the Japanese Genotype–phenotype Archive (JGA) with an accession code JGAS000653 for general research use, which can be accessed through the application at the NBDC with the accession code hum0419. For any additional relevant data, interested parties may obtain access directly from the authors. After the publication of this paper, we intend to make PDT-LUAD#99 tumoroids available through the RIKEN Cell Bank (https://cell.brc.riken.jp/en/).

## Declarations

### Additional information

Correspondence and requests for materials should be addressed to T.F.
