# Supplementary figures and images for: Establishment and characterization of novel high mucus-producing lung tumoroids derived from a patient with pulmonary solid adenocarcinoma
Source: Hum Cell. 2024 Apr 17;37(4):1194–204. doi: 10.1007/s13577-024-01060-3 (PMC11194211; doi:10.1007/s13577-024-01060-3)

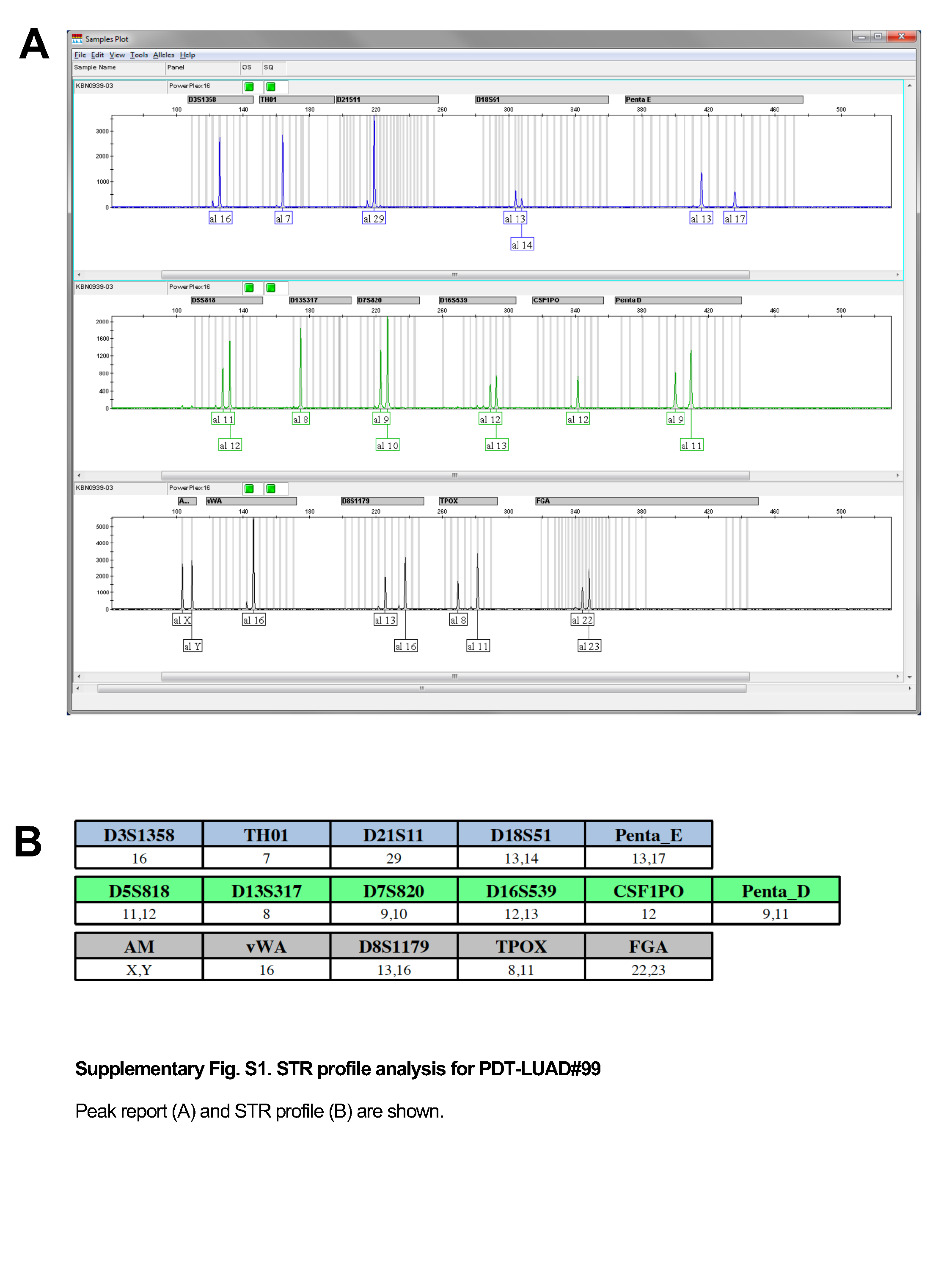

Supplement: Supplementary file 1 — Supplementary file1 (TIF 982 KB) [file 13577_2024_1060_MOESM1_ESM.tif]
